# Analysing the interplay of environmental virology, public health, and sanitation: a comprehensive review from a Kenyan perspective

**DOI:** 10.3389/fcimb.2023.1256822

**Published:** 2023-10-24

**Authors:** Michael Wasonga Opere

**Affiliations:** ^1^ School of Pure and Applied Sciences, Kenyatta University, Nairobi, Kenya; ^2^ School of Biosciences, The University of Nottingham, Nottingham, United Kingdom

**Keywords:** viral threats, public health challenges, sanitation, waste management, environment, waterborne diseases

## Abstract

This comprehensive review examines the interplay between environmental virology, public health, and sanitation in the unique context of Kenya. The review sheds light on the specific viral threats faced by the country, including waterborne viruses, zoonotic infections, and emerging viral diseases, and their implications for public health. It explores the prevailing public health challenges in Kenya associated with environmental viromics, such as infectious viral diseases, and the rising burden of other infectious particles. The role of sanitation in mitigating viral infections is highlighted, emphasising the importance of clean water supply, proper waste management, and hygienic practises. The review also presents strategies for strengthening environmental virology research in Kenya, including enhancing laboratory capacities and leveraging technological advancements. Furthermore, the policy implications and recommendations derived from the review emphasise the need for multi-sectoral collaboration, evidence-based decision-making, and long-term investments in infrastructure and behaviour change interventions. Implementing these strategies can enhance the understanding of environmental virology, improve public health outcomes, and ensure sustainable sanitation practises in Kenya, ultimately contributing to the well-being of the population and sustainable development.

## 1 Introduction

**Figure 1:**
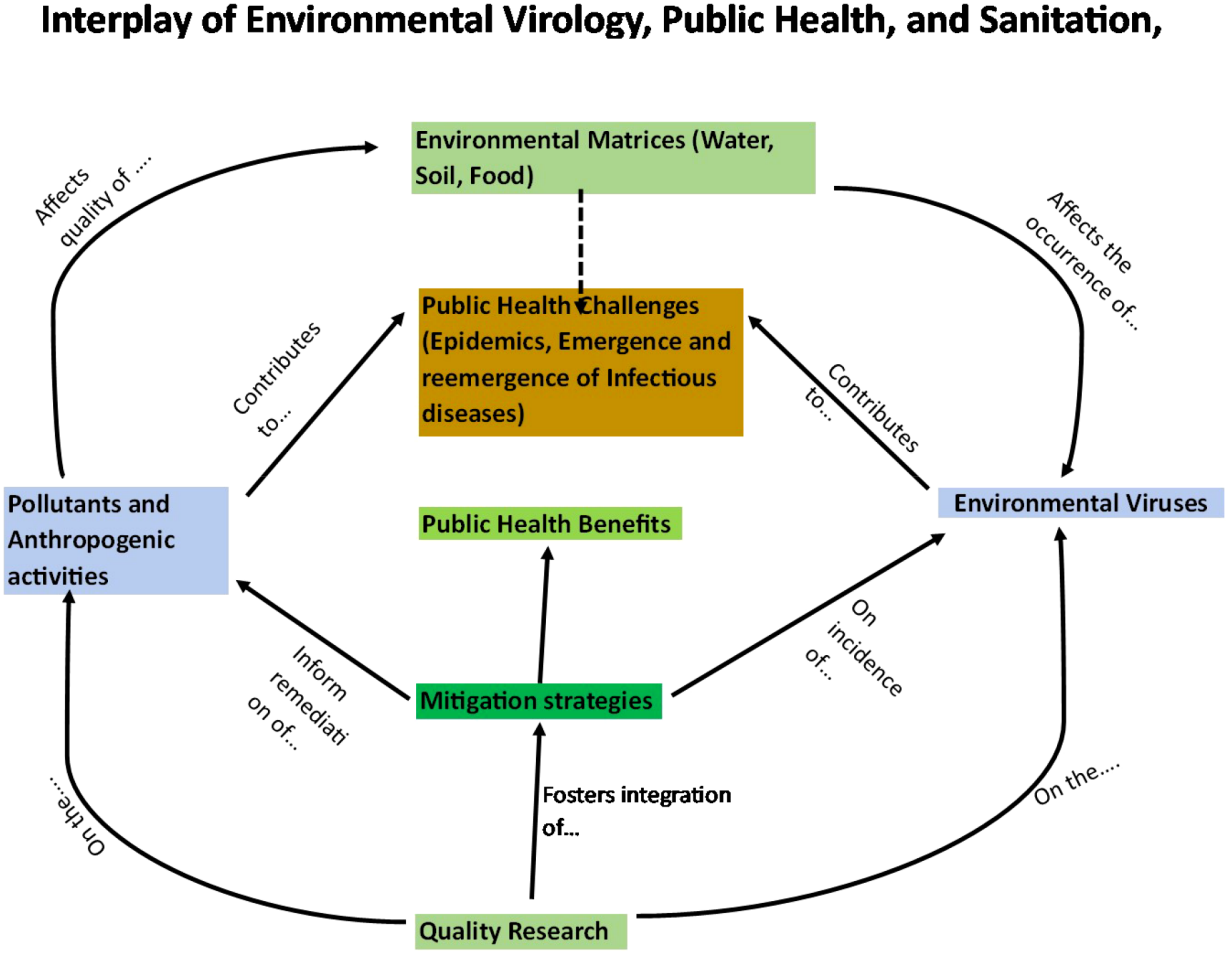
Schematic illustration on the connections among environmental virology, public health and sanitation.

The intricate interplay among environmental virology, public health, and sanitation assumes a pivotal role in mitigating the formidable challenges encountered by nations globally. In the Kenyan context, a nation grappling with substantial and diverse environmental and public health complexities, elucidating, and enhancing this interplay becomes of paramount significance. The overarching objective of this comprehensive review is to scrutinize the intricate dynamics between environmental virology, public health, and sanitation within Kenya, with a focal point on discerning the specific challenges and prospects within this domain. Kenya, akin to numerous African nations, confronts a spectrum of environmental and public health dilemmas, prominently inclusive of waterborne diseases (Bitanihirwe et al., 2021). The intricacy of the interrelationship is illustrated by **Figure 1**.

For example, rotavirus infections stand as a substantial contributor to diarrheal incidence in Kenya, particularly affecting the pediatric demographic (Muendo et al., 2018). Furthermore, Kenya has grappled with outbreaks of other viral diseases such as Rift Valley Fever (Ahmed et al., 2021) and COVID-19, which have exerted profound impacts on public health and the general welfare of the populace. Environmental virology, encompassing the examination of viral pathogens in the environment and their repercussions on human health, serves as the bedrock of this intricate nexus. By investigating the transmission, persistence, and impact of viral infections in the Kenyan context, we endeavor to glean insights into the distinctive challenges and opportunities arising from the nation’s diverse ecosystems, climatic patterns, and demographic dynamics.

Presently, environmental virology research in Kenya predominantly centers on the identification and characterization of viral pathogens within various environmental matrices, including water sources and wastewater. These studies employ molecular techniques such as polymerase chain reaction (PCR) to discern and quantify viral DNA or RNA. Nevertheless, a need persists for more comprehensive inquiries that delve into the enduring viability of viral pathogens under diverse environmental conditions and their potential modes of transmission to humans (van Zyl et al., 2019).

In tandem with environmental factors, socio-economic determinants wield considerable influence over public health outcomes in Kenya. Restricted access to healthcare, education, and resources compounds the burden of diseases, impairing the efficacy of prevention and treatment strategies. Additionally, cultural practices and beliefs can impact health-seeking behaviors and contribute to viral infection transmission. For instance, in specific regions, improper fecal disposal practices such as open defecation may heighten the risk of diseases such as adenoviruses. Tackling these socio-economic factors constitutes a pivotal step toward ameliorating public health in Kenya (Wasonga et al., 2020).

Research demonstrates that access to clean water, effective waste management, and hygienic practices constitute indispensable elements in curtailing the transmission of viral infections. For instance, studies underscore that the enhancement of sanitation infrastructure correlates with a significant reduction in waterborne disease incidence. Furthermore, the promotion of behavior change, exemplified by thorough handwashing with soap, has demonstrated efficacy in mitigating the spread of viral infections. These findings underscore the imperativeness of investing in sanitation measures to safeguard public health and advance sustainable development in Kenya (van Seventer and Hochberg 2017).

In recent years, Kenya has made substantial advancements in fortifying sanitation infrastructure and fostering behavior change. County governments and non-governmental organizations, including UNICEF, have invested in the construction of clean water sources and sanitation facilities in underserved regions, guaranteeing access to potable water and proper waste management (UNICEF, 2023). Moreover, community-based initiatives have been deployed to raise awareness regarding hygiene practices and facilitate behavioral transformations at the individual and household levels. These endeavors have yielded promising outcomes, substantially diminishing the incidence of waterborne diseases and enhancing public health.

This comprehensive review commences by surveying the current landscape of environmental virology research within Kenya, inclusive of viral pathogen identification in the environment and their implications for human health. Subsequently, it delves into the gamut of public health challenges confronting the nation, with a particular emphasis on waterborne diseases and emerging viral outbreaks. The review further expounds on the contemporary quandaries pertaining to environmental and public health virology within Kenya. Ultimately, it culminates by encapsulating the salient challenges and prospects inherent in the interplay among environmental virology, public health, and sanitation within the Kenyan context.

## 2 Data collection

A thorough literature review was conducted to gather relevant scholarly articles, research papers, reports, and case studies related to environmental virology, public health, and sanitation in the Kenyan context. Databases such as PubMed, Google Scholar, and relevant institutional websites were searched using keywords related to the topic. The gathered literature was carefully screened to select research articles and sources that provided significant insights into the interplay of environmental virology, public health, and sanitation in Kenya. The selected data were analysed and synthesised to identify key themes, trends, challenges, and opportunities. The review was organised into sections to provide a logical flow of information. These sections included the introduction, environmental virology in Kenya, public health challenges in Kenya, the role of sanitation in mitigating viral infections, strategies for strengthening environmental virology research, policy implications, and recommendations. Throughout the review, specific attention was given to incorporating the Kenyan perspective. This involved considering the unique environmental, socio-economic, and cultural factors relevant to Kenya’s public health and sanitation landscape. Examples, case studies, and data specific to Kenya were utilised to contextualise the discussions. The review draws upon the expertise and knowledge of researchers familiar with environmental virology, public health, and sanitation. The information provided is based on the existing body of knowledge and research up until the knowledge cutoff date in June 2023.

## 3 Introduction to environmental viral communities

This section briefly describes the key groups of viruses that are specific to, or important to, aquatic environments with public health ramifications. The list is not exhaustive, and the discussed groups are not endemic to Kenyan settings but are examples drawn from general publications to illustrate the general contexts. The viruses include Enteroviruses, a genus of positive sense ssRNA viruses that belong to the order Picornavirales (Quaranta et al., 2020) They can cause diseases such as poliomyelitis, meningitis, hepatitis, hand-foot-and-mouth disease, and respiratory infections (Aswathyraj et al., 2016). Enteroviruses can be transmitted through the faecal-oral route, respiratory droplets, or contact with contaminated water (Wells and Coyne, 2019). Hepatoviruses: a genus of positive sense ssRNA viruses that belong to the order Picornavirales (Le Gall et al., 2008). They can cause hepatitis A, an acute liver infection that can be spread through the ingestion of contaminated food or water (Jacobsen, 2018). Rotaviruses: a genus of dsRNA viruses that belong to the family Reoviridae. They can cause severe gastroenteritis, especially in children, and can be transmitted through the faecal-oral route or contact with contaminated water (Le Gall et al., 2008).

Adenoviruses: Adenoviruses are a group of viruses that can infect different organs and tissues in humans and animals. Adenoviruses have a double-stranded DNA genome and a non-enveloped icosahedral capsid. [Adenoviruses are one of the groups of enteric viruses, meaning they can be transmitted through the faecal-oral route, but they can also be spread by respiratory droplets or contact with infected surfaces Adenoviruses can cause various diseases, such as respiratory infections, conjunctivitis, gastroenteritis, cystitis, and obesity (Lischka, 2022)].

Noroviruses: Noroviruses are a group of viruses that belong to the family Caliciviridae (Karol et al., 2021). They cause gastroenteritis, an inflammation of the stomach and intestines (Karol et al., 2021). They are also known as the winter vomiting bug or the stomach bug (Sion et al., 2023). Noroviruses have a single-stranded positive-sense RNA genome and a non-enveloped icosahedral capsid (Vinjé et al., 2019). Astroviruses: Astroviruses are a group of viruses that belong to the family Astroviridae and cause gastroenteritis, an inflammation of the stomach and intestines. They are also known as the stomach bug or the star-like virus because of their shape (Vinjé et al., 2019). Astroviruses have a single-stranded positive-sense RNA genome and a non-enveloped icosahedral capsid.

Sapoviruses: Sapoviruses are a type of small, single-stranded RNA virus that belong to the Caliciviridae family. They are known to cause gastroenteritis, an inflammation of the stomach and intestines characterised by symptoms such as diarrhoea, vomiting, abdominal pain, and sometimes fever (Aswathyraj et al., 2016). Sapoviruses are transmitted primarily through the faecal-oral route, which means they can spread through contaminated food, water, or surfaces (Oka et al., 2015).

Cycloviruses: are an emerging group of viruses that belong to the family Circoviridae and have circular single-stranded DNA genomes. They have been found in a wide range of hosts, such as bats, rodents, birds, insects, and humans (Rosario et al., 2017). Cycloviruses can cause various diseases, such as gastroenteritis, respiratory infections, hepatitis, and neurological disorders (Rosario et al., 2017). However, the pathogenicity and transmission of cycloviruses are not well understood (Dennis et al., 2018).

## 4 Regional studies of environmental virology and public health

The summary of select studies from the East African neighbouring countries that have investigated the environmental viral community in the last decade is summarised in **Table 1**.

**Table 1:** Select regional environmental virology and public health studies in the last decade.

Matrix	Location	Virology	Key Experimental Details	Reference
Vector borne	South Omo, Southern Ethiopia	Yellow fever and chikungunya	Serological assays	(Endale et al., 2023)
Hospital based	Mulago Hospital, Uganda	Rotavirus	Serological analysis	(Nakawesi et al., 2010)
Vector borne	Kenya	arboviruses	cell culture; RT-PCR screening, and sequencing.	(Ochieng et al., 2013)
Hospital based; animal-based	Kampala Uganda	Rotavirus	Next Generation Sequencing	(Bwogi et al., 2016)
Hospital/Animals	Masaka Uganda	Rotavirus	Virological assays	(Bwogi et al., 2023)
Hospital based	Dar es Salaam, Tanzania	Adenovirus	Serological assays; Virological assays	(Moyo et al., 2014)
Hospital based	Bagamoyo, Tanzania	enterovirus, adenovirus, and rotavirus	A matched case-control study design	(Mattioli et al., 2014)
Hospital based	Awassa, southern Ethiopia	Norovirus and rotavirus	Serological assay; Virological assay	(Yassin et al., 2012)
Animal focused	Bishoftu, Ethiopia	Sapoviruses and Noroviruses	Virologial assay	(Sisay et al., 2016)
Hospital based	Kampala, Uganda	Adenovirus	Virological assay	(Ukuli et al., 2023)
Surface water	Kamapala Uganda	adenovirus, enterovirus, rotavirus, and hepatitis A virus	Virological assay, next-generation sequencing	(O'Brien et al., 2017)
surface water, grey water, and ground water	Kampala, Uganda	human adenoviruses F and G, Rotavirus, Hepatitis A virus, Hepatitis E virus, and human adenovirus species A,C,D,E, and F (HAdV-ACDEF)	Virological assay	(Katukiza et al., 2013)

These studies give the Kenyan data context for interpretation. The environmental viral community studies are typically dominated by rotaviruses, just like in the Kenyan studies (Kiulia et al., 2010). However, in some cases, a community equally dominated by adenoviruses (types 40 and 41) and enteroviruses (Opanda et al., 2016) was reported. (Haramoto et al., 2018) found that the most abundant class of enteric viruses was different on different types of matrices, indicating that systemic pollution influences community structure and composition. This supports the observation of (Wasonga et al., 2021) and suggests that the matrix with high faecal pollution levels and minerology influences the viral community composition. Even less research has been done on other viral families, like hepatoviruses, as indicators of faecal pollution than on other environmental viruses and bacteria. Hepatitis E was examined in one study in developing nations. Socioeconomic conditions, sanitation standards, access to potable water, and the regional occurrence of zoonotic HEV infections in animals were some of the factors that infection patterns were linked to (Khuroo et al., 2016). With the exception of sapoviruses, caliciviruses have also not been thoroughly studied.

## 5 Introduction to environmental viral communities in Kenya and the public health challenges

Some significant environmental viral studies have been undertaken in Kenya that present data on the environmental virology of different epidemiological aspects (**Table 2**). In one study, the frequency of different viral communities’ contamination from diverse matrices was all above 97% (Lambisia et al., 2023).

**Table 2:** Select environmental virology studies in Kenya.

Epidemiology	Location	Virology	Experimental Details	Number of samples (n)	Positive samples (%)	Reference
Food handlers	Nairobi County, Kenya	Noroviruses	Molecular assays	283	43 (15.2	Wainaina et al., 2020
Surface water	Homa Bay County, Kenya	Adenovirus	Molecular assay	216	7 (3.2)	(Wasonga et al., 2020)
Surface water	Homa Bay County, Kenya	Enterovirus	Molecular assays	216	11 (5)	(Wasonga et al., 2020)
Hospital based and House Holds	Kilifi County	Adenovirus	Hospital based	91	88 (97)	Lambisia et al., 2023
Hospital based and House Holds	Lwak (Siaya County), Kibera slums	Norovirus	RT-PCR	858	244 (28.4)	Shioda et al., 2016
Hospital based and House Holds	Lwak (Siaya County), Kibera slums	Sapovirus	RT-PCR	858	44 (5.1)	Shioda et al., 2016
Hospital based and House Holds	Lwak (Siaya County), Kibera slums	Astrovirus	RT-PCR	858	46 (5.4)	Shioda et al., 2016
Informal settlement setting	Kibera slums	Enteroviruses	Enteric Taqman array cards	628	209 (78)	Mwasi et al., 2019
Hospital based (Following vaccination introduction)	Kenyatta National Hospital, Nairobi	Rotavirus	Serological assay (enzyme linked immunosorbent immunoassay)	365	53(14.5)	Muendo et al., 2018

Studies from other regions of the country support the notion that changing the composition of some viral gene groups is a typical response to pollution, and a similar change in relative abundance has been observed in hydro-chemically contaminated areas of a freshwater matrix (Wasonga et al., 2021). However, because these observations are based on a small number of studies, it is challenging to predict with certainty the changes that will probably be seen in other environmental matrices and settings. The various results of these investigations, however, imply that microbial communities might respond to pollution in various and site-specific ways. To understand these viral communities, a much broader examination of all environmental viruses in Kenyan environmental matrices is needed since there are still relatively few studies on viral communities.

What is certain, though, is that the few studies presented here have shed light on the impending public health issues related to environmental virus contamination. Public health is faced with a wide variety of viral threats, which calls for a deeper comprehension of environmental virology. For instance, waterborne viral infections are a serious problem in Kenya, especially in areas with poor access to clean water and inadequate sanitary facilities (Adelodun et al., 2021). Particularly in vulnerable populations like children and those with weakened immune systems, waterborne viral infections increase the burden of diarrheal diseases. To implement efficient preventive measures and guarantee access to safe drinking water, it is essential to comprehend the origins, modes of transmission, and persistence of these waterborne viruses.

Kenya’s rich biodiversity and close interactions between humans and animals create favourable conditions for zoonotic infections. Diseases such as avian influenza and Rift Valley fever have zoonotic origins and can be transmitted from animals to humans (Sisay et al., 2016). In addition, Kenya, like any other developing country, is vulnerable to emerging viral diseases due to factors such as urbanisation, population growth, and increased international travel. Emerging diseases like the Zika virus, dengue fever, and chikungunya have gained attention in recent years. Knowledge in this area is vital for timely surveillance, early detection, and an effective response to emerging viral outbreaks. The emerging viral diseases have the potential to spread rapidly, leading to increased morbidity and mortality rates. Therefore, understanding the environmental virology aspects of these diseases can guide public health interventions and resource allocation to minimise their impact.

## 6 Contemporary issues in Kenya: environmental virology and public health

### 6.1 Poor sanitation standards

Poor sanitation is associated with open defecation, which can facilitate the spread of enteric viruses and other pathogens. A study found that open defecation decreased from 16.2% in 2003 to 9.9% in 2014, but the burden increased among poor households, especially the poorest. The study also found disparities in access to improved sanitation facilities between different wealth quintiles (Njuguna, 2019).

### 6.2 Water quality and availability

Water resources are threatened by deforestation, soil erosion, desertification, flooding, pollution, and climate change. These factors can affect the quantity and quality of water for domestic, agricultural, and industrial use, as well as the health of aquatic ecosystems.

### 6.3 Diffuse pollution

A common occurrence in Kenya is diffuse pollution, which can introduce or increase the concentration of viral pathogens in the water sources, putting the public at risk of exposure and infection. For instance, enteric viruses like norovirus and hepatitis A virus can be transported into water sources like rivers, dams, and marine estuaries by urban stormwater. The dynamics and epidemiology of waterborne viral diseases can be affected by diffuse pollution, which can also have an impact on the survival, persistence, and transmission of viral pathogens in water sources.

### 6.4 Climate change

The distribution and abundance of hosts and vectors of viral pathogens in water sources can change due to climate change, which can affect the risk and transmission of waterborne viral diseases. For instance, climate change may result in higher temperatures, more precipitation, and greater humidity. These factors may favour the survival of these vectors that transmit viruses. For example, one study demonstrated that orthobunyaviruses like Bunyamwera and Nyando, which can cause febrile illnesses with rash, hemorrhagic fever, and congenital malformations in both people and animals, can be transmitted by mosquito (Koka et al., 2021). Climate change can also increase the frequency and intensity of extreme weather events, such as floods, droughts, and storms, which can damage the infrastructure and facilities for water supply, sanitation, and wastewater treatment. This can result in the discharge or leakage of raw or inadequately treated sewage into water sources, contaminating them with enteric viruses, such as norovirus and hepatitis.

### 6.5 Pathogens transport

The potential impact of pathogens being transported into drinking water is arguably the most urgent concern regarding viruses in environmental matrices. The primary sources of pathogen contamination are sewage treatment and waste from farm animals. However, some viral pathogens have been detected in native wild animals; for example, rats, mice or birds (Duarte et al., 2019). Although these may not be significant disease reservoirs, it could be argued that pathogens (even in small numbers) could be thought of as a part of the indigenous transient community in environmental matrices due to the pathogens’ presence in native animals and their inevitable migration into waterways.

## 7 Strategies for strengthening future research needs

Monitoring viral pathogens in the environment and spotting potential public health risks require a strong surveillance system as well. To aid in timely detection and response, existing surveillance systems can be strengthened, and early warning mechanisms developed for viral threats. This entails setting up sentinel surveillance sites, incorporating real-time data sharing platforms, and incorporating environmental virology data into already-existing surveillance frameworks. Utilising technological advancements for detection is another crucially important strategy. Environmental virology research has new opportunities thanks to technological advancements. Viral detection, genetic characterization, and environmental monitoring can all be revolutionised by incorporating tools like metagenomic sequencing, remote sensing, and bioinformatics. Utilising these technologies can improve research’s effectiveness and efficiency while revealing important details about the dynamics of viruses, their patterns of transmission, and the environmental factors that affect viral infections in Kenya. A sensory technological revolution is currently happening in environmental monitoring as well as clinical diagnosis, resulting in a paradigm shift in microbiological and other contaminants analysis. This is one area where early warning is crucial to enhancing future research.

## 8 Conclusion

Despite significant strides in various aspects of environmental virology, our understanding of native virological communities within Kenyan environmental matrices remains rudimentary. To bridge this knowledge gap, it is imperative that future investigations delve deeper into the factors driving viral diversity and the constituents of these communities. Such endeavors hold global significance, as comprehensive studies in this domain are scarce. A nuanced comprehension of environmental virus diversity and its response to pollution could pinpoint areas necessitating heightened management and monitoring, while also identifying resilient or safeguarded communities amidst anthropogenic pressures. Only by comprehensively grasping the current state of environmental virology can we discern alterations stemming from potential future human impacts.

Among the paramount research priorities, delineating the overarching determinants influencing viral community structures across diverse Kenyan environmental contexts looms large. This foundational insight should serve as a compass guiding applied research endeavors, underpinning initiatives aimed at preserving environmental quality, devising bioremediation strategies, and tracking the transport of viral pathogens. This review has unveiled the spectrum of viral threats confronting Kenya, underscored persistent public health challenges, and elucidated the pivotal role played by sanitation in mitigating viral infections. It is my hope that this synthesis of knowledge paves the way for a more profound understanding of the complex interplay among environmental virology, public health, and sanitation, ultimately contributing to the betterment of both Kenyan and global communities.

## Author contributions

MO: Writing – original draft.
